# Evening home lighting adversely impacts the circadian system and sleep

**DOI:** 10.1038/s41598-020-75622-4

**Published:** 2020-11-05

**Authors:** Sean W. Cain, Elise M. McGlashan, Parisa Vidafar, Jona Mustafovska, Simon P. N. Curran, Xirun Wang, Anas Mohamed, Vineetha Kalavally, Andrew J. K. Phillips

**Affiliations:** 1 School of Psychological Sciences and Turner Institute for Brain and Mental Health, Monash University, Melbourne, VIC Australia; 2 Department of Electrical and Computer Systems Engineering, School of Engineering, Monash University Malaysia, Bandar Sunway, 47500 Subang Jaya, Malaysia

**Keywords:** Circadian rhythms and sleep, Circadian mechanisms, Circadian regulation, Sleep

## Abstract

The regular rise and fall of the sun resulted in the development of 24-h rhythms in virtually all organisms. In an evolutionary heartbeat, humans have taken control of their light environment with electric light. Humans are highly sensitive to light, yet most people now use light until bedtime. We evaluated the impact of modern home lighting environments in relation to sleep and individual-level light sensitivity using a new wearable spectrophotometer. We found that nearly half of homes had bright enough light to suppress melatonin by 50%, but with a wide range of individual responses (0–87% suppression for the average home). Greater evening light relative to an individual’s average was associated with increased wakefulness after bedtime. Homes with energy-efficient lights had nearly double the melanopic illuminance of homes with incandescent lighting. These findings demonstrate that home lighting significantly affects sleep and the circadian system, but the impact of lighting for a specific individual in their home is highly unpredictable.

The circadian system is fundamental for human health^1^. The most important environmental time cue for the circadian system is light, with the effects mediated primarily by the photopigment melanopsin^2–4^. In our ancestral past, the circadian system received strong on/off signals, with bright days and dark nights^5,6^. The availability of electric lighting, coupled with our modern indoor lifestyle, has profoundly changed how humans interact with light. Exposure to light at night suppresses production of the sleep-promoting hormone melatonin^7^ and causes circadian disruption, which is associated with a range of poor health outcomes, including disrupted sleep^8^.

Traditional incandescent lights expend a large percentage of their used energy as heat, making them energy inefficient. For this reason, there has been a global effort to transition from incandescent lights to more energy-efficient LED and compact fluorescent lights^9,10^. As a demonstration of the impact of energy-efficient lighting, the 2014 Nobel Prize in Physics was awarded "for the invention of efficient blue light-emitting diodes which has enabled bright and energy-saving white light sources." As white LED lights are typically enriched in blue light, this global transition could exacerbate circadian disruption and consequent sleep disturbance due to a greater impact of energy-efficient lighting on short wavelength-sensitive melanopsin^11^.

Recent evidence has revealed that the human circadian system is more sensitive to evening light than previously thought^2,12,13^, and that there are substantial interindividual differences in light sensitivity^12^. These interindividual differences are especially pronounced at indoor light levels^14^. However, methods used to measure real-world light environments to date have not accurately assessed the impact of light on the circadian photopigment melanopsin. Retinal light exposure is the dominant synchronizer of the human circadian system^15^. Most wearable light sensors used in research are wrist-worn, prone to sleeve coverage, and do not capture light exposure at eye level^16^. The real-world significance of interindividual differences in light sensitivity is therefore yet to be determined. To address this, we developed a new wearable spectrophotometer that measures light near eye level and calculates the impact of light on melanopsin. We measured objective sleep quality and light exposure patterns in freely-living adults. Using individual-level dose-response curves to light, we also estimated the differential effects of home lighting on the human circadian system.

## Results

### A wearable spectrophotometer

**Figure 1:**
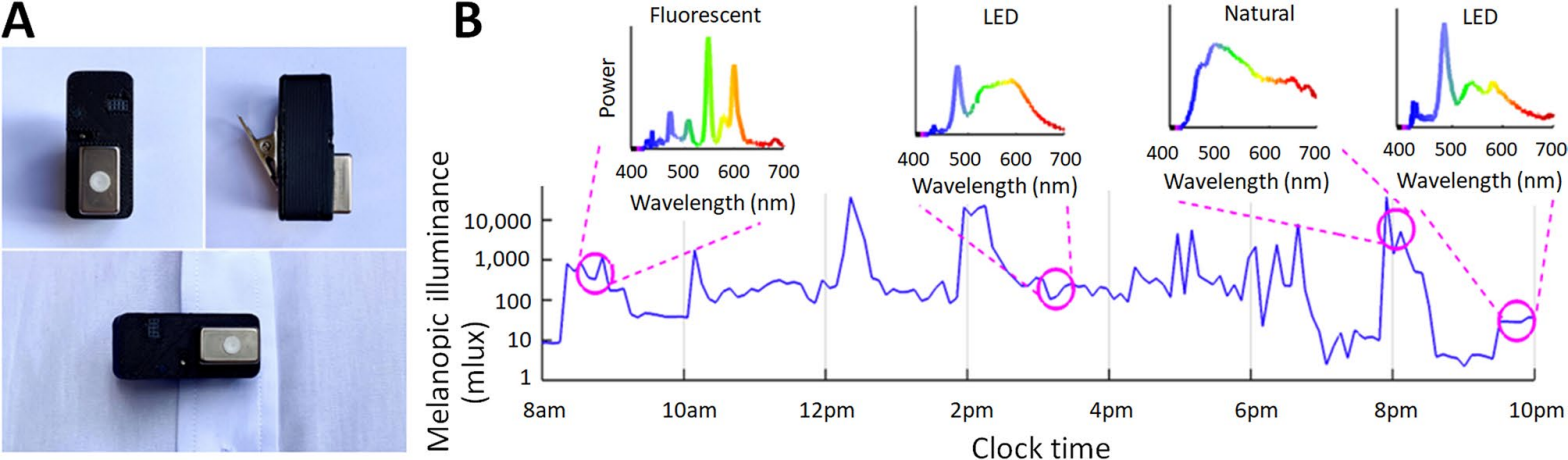
A new wearable for assessing ambulatory melanopic illuminance. (**A**) The wearable spectrophotometer is clipped to clothing and records light exposure near eye level. (**B**) An example of melanopic illuminance across a single waking day, with pop-outs showing recorded spectra at certain times of day.

We developed a wearable device containing a mini-spectrophotometer that records the full spectrum of visible light (see “Methods”). From spectra, the device calculates the impact of light for each photoreceptive input in the human eye, including the impact of light on the circadian photopigment melanopsin (melanopic illuminance)^3, 17,18^, as well as the impact of light on photopic vision (photopic illuminance). Furthermore, the spectral signatures allow determination of the type of light source. Figure 1 shows the device and melanopic illuminance (mlux) across a waking day.

### Individual differences in circadian light sensitivity intersect with variable home lighting

Recently, we discovered that there are over 50-fold differences in the sensitivity of the human circadian system to light between individuals^12^. The light level required for 50% melatonin suppression (ED50) ranged from 3.1 to 181 mlux (using updated CIE standards^19^) between individuals (n = 42). This range of ED50 values overlaps with the observed range of home lighting levels we found. Average melanopic illuminance in the 3 h before bedtime ranged from 3.9 to 77.4 mlux between homes (M = 17.9 mlux, SD = 13.6 mlux; n = 59 homes). This implies that, between people, the circadian system would respond very differently to the same home light environment.

**Figure 2:**
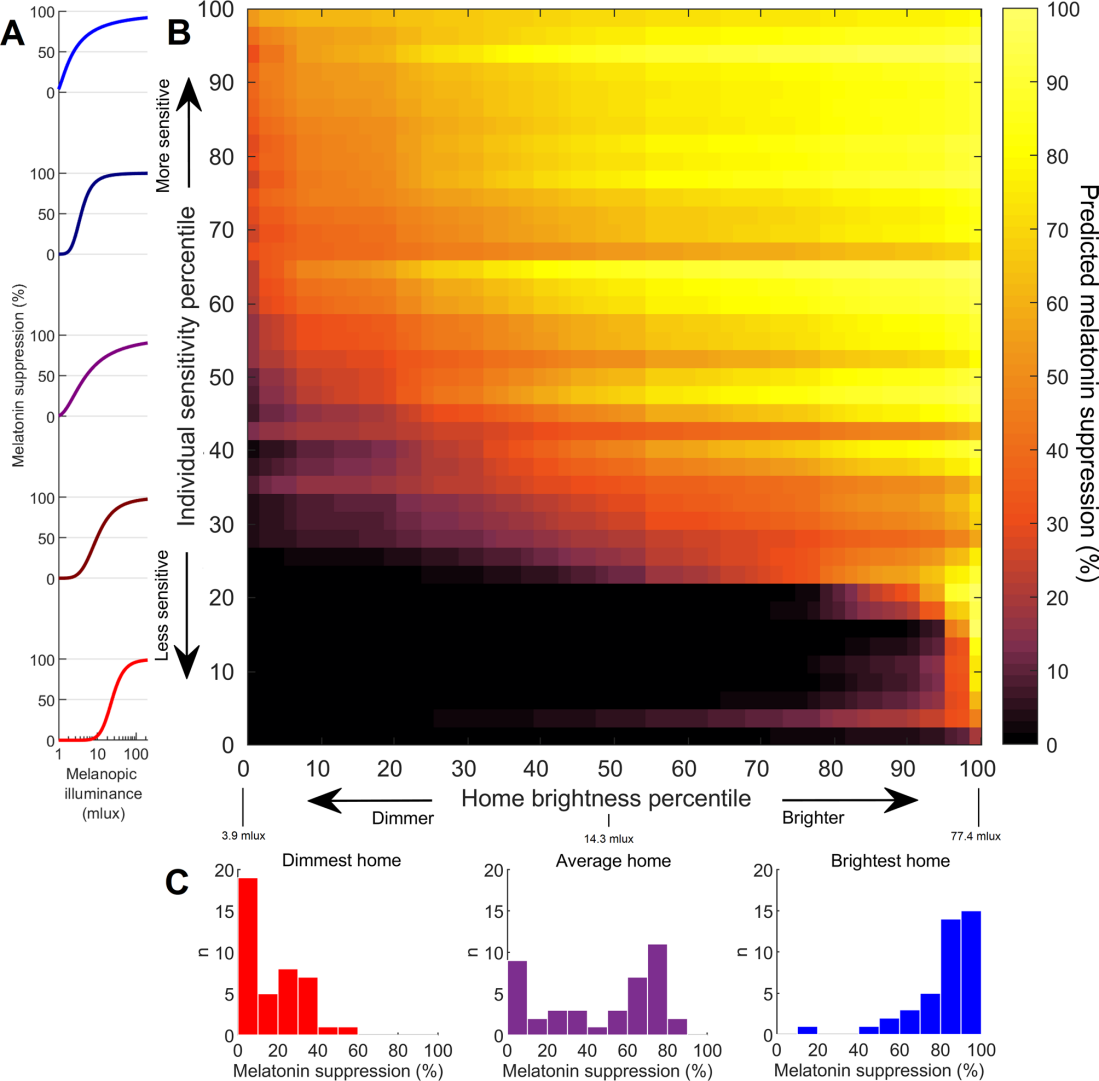
Home lighting in the context of circadian light sensitivity. Using individual-level dose-response curves for light (**A**) in combination with home light recordings, we predicted melatonin suppression in each home across the range of individual sensitivity levels (**B**), with rows sorted by number of homes with > 25% suppression. Predicted responses across individuals are shown as histograms (**C**) for the dimmest home, average home (median brightness), and brightest home.

By combining our physiological measures of circadian light sensitivity with our detailed evening light recordings, we predicted the impact of home lighting on the circadian system. Using individual-level dose-response curves to evening light, we calculated percentage melatonin suppression for each home (Fig. 2). We found that 48% of homes were predicted to cause at least 50% melatonin suppression, averaging across individual sensitivity levels. Furthermore, 73% of homes were predicted to cause at least 20% melatonin suppression, while 15% of homes were predicted to cause at least 80% melatonin suppression.

We found vastly different predicted responses to the same home light environment between individuals (Fig. 2B). For the most sensitive individual (top row of Fig. 2B), 100% of homes were predicted to cause at least 50% melatonin suppression, whereas for the least sensitive individual (bottom row of Fig. 2B), 0% of homes were predicted to cause at least 50% melatonin suppression. The average home resulted in a wide range of predicted individual responses, from 0 to 87% melatonin suppression (Fig. 2C). Our findings demonstrate that the circadian impact of lighting for any given individual in any given home is highly unpredictable without knowing both their individual level of sensitivity and home lighting levels.

**Figure 3:**
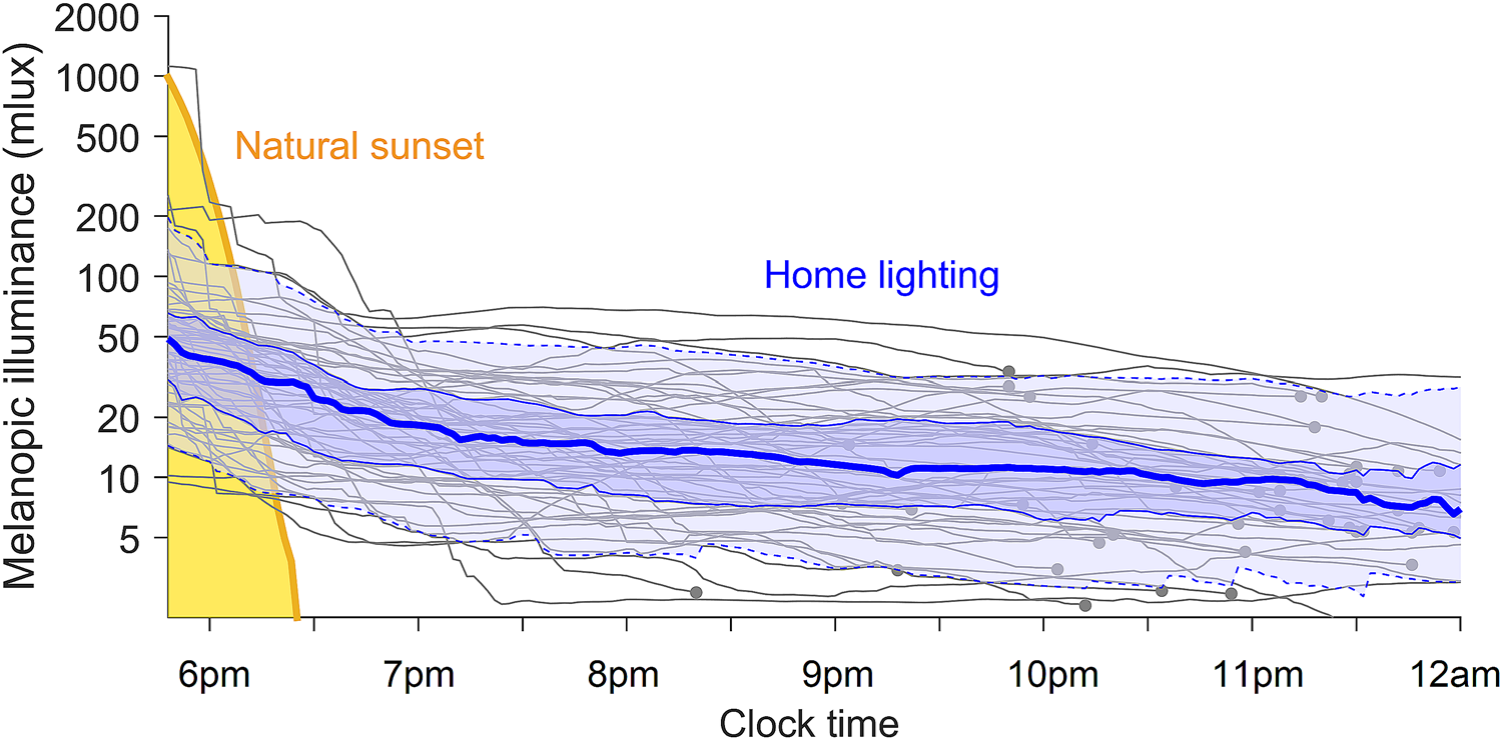
Artificial light results in an extended twilight. Changes in melanopic illuminance in the evening for a natural sunset (yellow line), compared with home lighting. Individual homes are shown as gray curves, averaged across nights and smoothed with a 3-h moving average, ending with a gray dot at the individual’s average bedtime. The blue lines show the median (thick solid line), interquartile range (thin solid lines), and the 10th and 90th percentiles (dashed lines) for homes before bedtime.

To evaluate how home light differed from natural light, we measured sunset using our wearable spectrophotometer (single recording with daylight duration of 11:13). Natural light levels dropped rapidly from the level needed for 50% melatonin suppression in the least sensitive individual (ED50 = 181 mlux) to the most sensitive individual (ED50 = 3.1 mlux) in 22 min (Fig. 3). In modern homes, we spend more time in this range, where interindividual differences in the response of the human circadian system to light are most pronounced. We found that home lighting persisted at biologically impactful levels throughout the evening (Fig. 3). At 8 pm (n = 59 pre-bed recordings), median melanopic illuminance was 13.4 mlux (range 2.5–67.8 mlux, M = 16.9 mlux, SD = 12.7 mlux). At 10 pm (n = 50 pre-bed recordings), median melanopic illuminance was 11.0 mlux (range 2.4–49.6 mlux, M = 13.0 mlux, SD = 9.1 mlux). These levels are comparable to the group-average ED50 (12.7 mlux)^12^, as well as the median individual ED50 (14.0 mlux). These findings indicate that home lighting creates an extended twilight for the circadian system, weakening the distinction between day and night.

### Greater evening light exposure is associated with poorer sleep

Since light exposure before bed has been shown to disrupt the first sleep cycle under laboratory conditions^20^, we investigated whether evening light exposure related to objective sleep quality in the field. We found that increased melanopic illuminance, relative to an individual’s average, in the 3 h before bedtime was associated with increased wakefulness for that individual in the 90 min after bedtime (Table 1; β = 0.89 ± 0.09, p < 0.00001). This relationship held when adjusting for age, sex, average bedtime, and deviation from average bedtime (Table 1). The relationship also held (Table S1; β = 0.89 ± 0.09, p < 0.00001) when adjusting for age, sex, average bedtime, deviation from average bedtime, chronotype (Morning-Eveningness Questionnaire score), Insomnia Severity Index, Epworth Sleepiness Scale, and the Pittsburgh Sleep Quality Index. An individual’s average melanopic illuminance across evenings was not predictive of their amount of wakefulness in the 90 min after bedtime (p = 0.59). This is consistent with the large interindividual differences we found in light sensitivity, as the effect of an individual’s average light level would be highly dependent on that individual’s light sensitivity.

**Table 1:** Within-individual variation in pre-bedtime light exposure is associated with objective sleep quality.

Wakefulness (minutes) in first 90 min after bedtime						
Within and between individual light, adjusted for age, sex, and bedtime						
Predictor	β	SE	t	Lower	Upper	p
Average light	− 0.42	0.62	− 0.69	− 1.64	0.79	0.49
Deviation from average light	0.89	0.09	9.88	0.71	1.07	** < 0.00001**
Age	− 0.03	0.01	− 2.59	− 0.046	− 0.006	**0.01**
Female sex	− 0.42	0.31	− 1.34	− 1.04	0.20	0.18
Average bedtime	0.55	0.49	1.13	− 0.41	1.52	0.26
Deviation from average bedtime	− 0.01	0.03	− 0.51	− 0.07	0.04	0.61

Generalized linear mixed model results for minutes of wakefulness in the first 90 min after bedtime, using a binomial distribution and logit link function. Predictors included age (years), sex, average light (log-transformed melanopic illuminance in 3 h before bedtime), deviation from average light (using log-transformed values), average bedtime (decimal hours), and deviation from average bedtime (hours). The table shows unstandardized coefficients (β), standard errors (SE), t value, Lower and Upper 95% confidence intervals, and p-values (significant values in bold).

### Melanopic illuminance is higher in homes with energy-efficient lighting

We computed an average spectrum for each individual night using light recordings in the 3 h before bedtime. The average spectrum reflected the combination of all light sources when more than one type of light source was present. For each night, we calculated: (1) melanopic illuminance, the non-visual impact of light, and (2) the ratio of melanopic illuminance to photopic illuminance (M:P ratio), a ratio of non-visual to visual impact.

**Figure 4:**
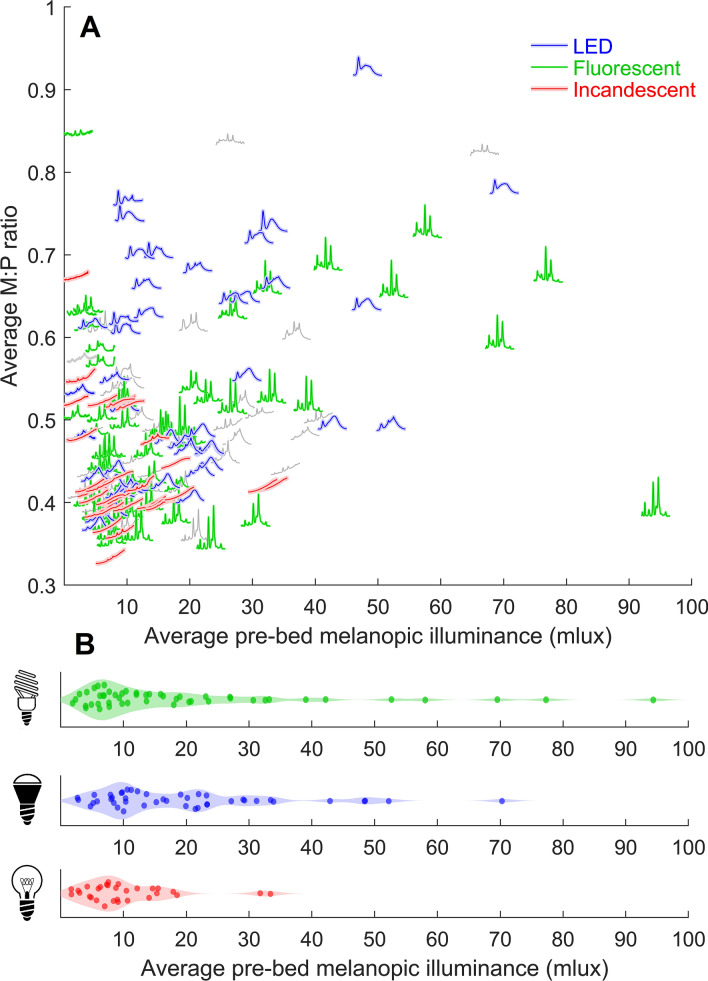
Energy-efficient lighting is associated with higher melanopic illuminance. (**A**) Average spectra for each night (3 h up to bedtime) are plotted according to average melanopic illuminance (x-axis position) and melanopic-to-photopic ratio (M:P ratio; y-axis position). Spectra that were classified as predominantly one light source type are colored green (fluorescent), blue (LED), or red (incandescent). Spectra that were unclassified/hybrids of light types are colored gray. (**B**) Distributions of melanopic illuminance for the three light types.

Key spectral characteristics for common types of light sources (incandescent, fluorescent, LED, and sunlight) were used to categorize the average spectrum for each night. In total, 76% of nights (n = 124) had one clearly predominant type of light source, while 24% of nights (n = 39) were unclassified/hybrids of light types. Of the nights with one clearly predominant type, 50 were fluorescent, 44 were LED, and 30 were incandescent (Fig. 4). Nights with incandescent lights had significantly lower average melanopic illuminance (10.3 ± 7.6 mlux) than nights with either LED lights (19.7 ± 14.9 mlux, p = 0.003) or fluorescent lights (19.7 ± 20.2 mlux, p = 0.01), and had significantly lower M:P ratio (0.44 ± 0.07) than LED lights (0.55 ± 0.14, p = 0.002) but not fluorescent lights (0.50 ± 0.11, p = 0.23). There was a large range of melanopic illuminance levels for both fluorescent (1.9–94.4 mlux) and LED lights (2.6–70.3 mlux), but a narrower range for incandescent lights (1.6–33.4 mlux). These findings indicate that the global transition to more energy-efficient lighting and the phase-out of incandescent lighting is resulting in a greater impact of home lighting on the human circadian system.

## Discussion

In this study, we used a new wearable spectrophotometer to assess the non-visual impacts of light exposure in modern homes on sleep and the circadian system. Some individuals are far more vulnerable to effects of light than others, and these interindividual differences are exacerbated by our ability to self-select light exposure in the evening. We found that light levels were highly variable between homes, with a 20-fold range in average melanopic illuminance in the 3 h leading up to bedtime. Due to the over 50-fold range in individual light sensitivity, the degree of melatonin suppression was predicted to vary greatly between individuals. The interplay of a 20-fold range in home lighting with an over 50-fold range in light sensitivity means that predicting the impact of an individual’s home lighting choices on their circadian system is highly challenging. This unpredictability differs from the highly predictable effects of the natural light/dark cycle on the circadian system. The variations we observed in home light levels and light sensitivity help to explain why early and late sleepers converge in both their sleep and circadian timing when taken away from variable home lighting and exposed to the same natural light/dark cycle^21^—conditions that are predicted by mathematical modeling to reduce interindividual differences in sleep and circadian timing^22,23^.

Although melanopic illuminance in homes varied greatly, we found that the most sensitive individuals would be highly impacted (> 50% melatonin suppression) by even the dimmest homes. The average home would suppress melatonin by nearly 50% in the average person. Given that we studied light sensitivity in healthy adults, there is potentially even greater impact of home lighting for individuals with heightened circadian light sensitivity, such as children^24^, clinical populations (e.g., sleep^25^ and mood disorders^26,27^), or those taking medications that increase light sensitivity^28^. Greater light sensitivity would put individuals at potentially greater risk of circadian disruption, due to more labile circadian phase^29,30^. An important next step will be the generalization of accurate light tracking to include these populations. Adjustment of home lighting is a potential intervention pathway for mood and sleep disorders, which has not been carefully explored to date.

Individual differences in sensitivity may be shaped to some extent by an individual’s home lighting. Experimental studies have shown that recent bright light exposure causes desensitization of the melatonin suppression response to light^31,32^. Individuals who live in brighter homes could plausibly become less sensitive to light as a result, although we note that habitual light exposure patterns from wrist-worn actigraphy did not relate to individual sensitivity in our prior study^12^. Future work integrating physiological measures of light sensitivity with home lighting choices will be needed to clarify this possible relationship.

Greater melanopic illuminance in the 3 h before bedtime was associated with poorer sleep efficiency in the first 90 min after bedtime. This result is consistent with the finding that exposure to blue-enriched light before bedtime results in reduced slow-wave activity specifically in the first sleep cycle^20^. These findings could reflect a light-induced delay in the timing of the circadian clock, resulting in a later signal for sleep onset, and therefore lower sleep efficiency. Brighter pre-bedtime lighting relative to an individual’s average may also have been driven by a third factor (e.g., increased socialization or entertainment) that resulted in both disrupted sleep and increased light exposure. Alternatively, these findings could reflect the tendency for melanopsin to have a sustained response to light even after lights have been switched off^33,34^. In homes with greater melanopic illuminance, sustained activation of melanopsin could result in a persistent alerting signal, contributing to lower quality sleep. As the sustained activation of melanopsin shows interindividual differences^35,36^, this may be a previously unappreciated mechanism for poor sleep, particularly in some clinical groups.

Humans have a unique degree of control over the light environment. However, we often make poor health choices with our lighting due to low awareness of the powerful non-visual effects of light. The economic benefits of energy-efficient lighting are potentially outweighed by the substantial disease burden^37,38^ and lost productivity^39^ due to chronic light-induced sleep and circadian disruption^8^. We found that melanopic illuminance was approximately 90% greater on average in homes that had energy-efficient lighting (LED, fluorescent) than in homes with incandescent lighting. However, homes that used energy-efficient lighting spanned a wide range of light levels. Some of these homes employed energy-efficient solutions that were not highly impactful on the circadian system in the evening. This may reflect the availability of LEDs or fluorescents that are dimmable or have a lower M:P ratio (e.g., warm LEDs). These observations indicate it is possible using energy-efficient lighting to achieve an evening light environment with similar melanopic illuminance to traditional incandescent lighting. However, many homes did not appear to employ such measures. This suggests a need for broader education regarding the sleep and health impacts of light, as well as more accessible biologically conscious lighting solutions.

Disruption of the circadian system contributes to many disease states. However, we currently lack the tools needed to effectively translate this knowledge into better health outcomes. Designing environments and interventions that promote healthy circadian rhythms depends on the ability to accurately and easily measure the effects of light environments on the circadian system. Here, we implemented a new method for monitoring the non-visual effects of light to measure the associated changes in sleep and to predict the real-world effect of evening home lighting on melatonin levels. Such methods, in combination with assessments of individual light sensitivity, are likely to be part of a future suite of tools that will enable individualized circadian medicine.

## Methods

### Participants

All procedures were approved by the Monash University Human Research Ethics Committee (MUHREC) prior to commencement. Participants gave written informed consent and were reimbursed for their time. This study was in line with the standards set by the Declaration of Helsinki (revision #7), except for registration in a database.

#### Dose-response curve study

Melatonin suppression data are from 56 healthy young participants (described in Phillips et al.^12^; 29 women, 27 men; age: M = 20.8, SD = 2.6 years). Participants were free from any medical or psychiatric conditions and were not taking medications at the time of the study, including hormonal contraceptives. They had not engaged in shift work in the preceding 12 months, nor recently traveled across time zones. Participants had a mean bed time of 23:04 (SD = 44 min) and rise time of 7:04 (SD = 44 min) during the study. Dim-light melatonin onset occurred on average at 21:05 (SD = 76 min), 2.22 h before bedtime.

#### Light monitoring study

A total of 62 participants, aged 18–65 years (age: M = 41.4, SD = 15.6 years; 30 women, 32 men), were recruited from the community for measurement of habitual daily light exposure patterns. Participants had not engaged in night shift work for at least 3 months preceding the study and had not recently traveled across time-zones (~ 1 week delay per 2-h shift, up to a maximum of one month). During the data collection period, the average bed and rise times for the sample were 23:22 (SD = 74 min) and 7:48 (SD = 76 min), respectively. Three participants failed to record data on any evening. After cleaning, data for 163 evenings were available for analysis from 59 participants.

### Protocol

#### Dose-response curve study

Circadian light sensitivity was assessed via the generation of individual dose-response curves using melatonin suppression data. Full data collection procedures are described in Phillips et al.^12^. Participants completed a 6-week or 7-week protocol, during which they maintained a strict sleep–wake schedule monitored with wrist-worn Actigraphs (Actiwatch Spectrum Plus/2/L, Philips Respironics, PA, USA). Weekly assessments of salivary melatonin were conducted from 4 h prior to until 1 h after bedtime, with hourly samples. All participants completed a dark control (< 1 lux) for the assessment of baseline melatonin levels and dim light melatonin onset (DLMO), followed by one of six randomly generated light exposure sequences (including intensities of 10, 30, 50, 100, 200, 400, and 2000 photopic lux, with M:P ratio of 0.51, using the latest CIE recommendations for calculation of melanopic illuminance^19^).

#### Light monitoring study

The in-home light data were collected between April 15 and July 14, 2019, in Melbourne, Australia (Autumn–Winter). Natural daylight ranged in duration from 9:32 (solstice) to 11:08, and natural sunset time ranged from 17:07 (solstice) to 17:54. Data collection took place across up to 4 nights per participant. Participants were instructed to keep daily routines and sleep–wake times that were ‘usual’ for them during the data collection period. Participants wore an Actiwatch Spectrum Plus (Philips Respironics, PA, USA) and kept a sleep diary. Participants attached the wearable spectrophotomer to clothing during wake periods. The spectrophotometer was placed on a nearby surface during rest periods, or during periods where it could not feasibly be worn (e.g., while showering). Participants were instructed to wear the device attached to their clothing on the chest, within ~ 20 cm of the chin/shoulder, with the sensor in line with eye gaze when facing forward. Rest intervals were determined using a combination of sleep diary data and visual inspection in Actiware (Philips Respironics, PA, USA).

A single recording of a natural sunset was taken on a clear day in August 2019, in Bentleigh, Melbourne, Australia (37.9224° S, 145.0410° E). Natural daylight duration was 11:13 and natural sunset was at 17:57.

#### Wearable light-measuring device

We developed and tested a wearable spectrophotometer to allow us to measure effective illuminances in the field, including photopic and melanopic illuminance. The device (Fig. 1) measured 44 × 20 × 29 mm, weighed 20 g, and was designed to be attached to clothing near to eye level. The device contained a C12666MA mini-spectrometer (spectral range 340–780 nm) from Hamamatsu Photonics, chosen due to its compactness, relatively high spectral resolution (15 nm), and high dynamic range. It is hermetically sealed and performs array spectroscopy using an array of 256 CMOS pixels with a reflective blazed grating for light diffraction.

The wearable spectrophotometer was calibrated to compensate for sensor characteristics such as dark current, stray-light, detector saturation, and nonlinearity. The baseline signal was characterized with the sensor placed in complete darkness, and the data were used to predict the corrections for electronic offset and dark current for any given integration time of the sensor. Stray-light correction was performed using a coefficient-based method adapted from calibration methods used for array spectroradiometers based on CIE 233:2019^40^. We also implemented an automatic optimal gain setting algorithm to ensure that the signal-to-noise ratio was high for a wide range of light intensities, and additionally corrected the unresolved non-linearity of the sensor to achieve a high dynamic range. The spectral responsivity of the device was characterized and calibrated using a detector-based calibration approach wherein the CL500-A (Konica Minolta, Tokyo, Japan) was the standard spectroradiometer. Wavelength accuracy was characterized using the 16 channels of the Telelumen Light Replicator. The peak wavelengths of the sixteen channels were measured by the calibrated CL500-A. The error in wavelength measured by the device fell within ± 6 nm. To ensure the device’s spatial response was near to Lambertian (similar to the human eye), diffusing film (3M Diffuser Film 3635-70) was used in the sensor’s optical input slit.

Non-linearity in measurements with changing input light intensity and varying integration times was characterized and calibrated to achieve a dynamic range from 1 lux to 60,000 lux. Inter-device variability in the measurement range for typical indoor light levels was maintained below 5% for the range 4–30,000 lux, and errors were within 0.7 lux for the range 1–4 lux. Five light sources, consisting of commonly encountered light sources (LED, fluorescent, incandescent, and sunlight) and a CIE standard D65 light were measured with both the CL500-A and the wearable device to verify the spectral calibration of the wearable device (Figure S1). The normalized root mean squared error between the spectra measured for the five light sources was maintained under 0.12. Photopic and melanopic illuminance were calculated, and the percentage error was maintained below 7%.

### Analysis

#### Dose-response curve study

Individual-level dose-response curves were fit using melatonin suppression data, following a procedure described previously^12^. Salivary DLMO was determined for each participant from the baseline night using an absolute threshold of 4 pg/mL. Linear interpolation was used to determine the first threshold crossing time. DLMO values were obtained for 55 of the 56 participants. For each light exposure condition, melatonin suppression was calculated by comparing area under the curve from the time of baseline DLMO to the final melatonin assay with area under the curve over the same time range in the baseline condition. A four-parameter logistic model was fit, from which the half-maximum (ED50) values of melanopic illuminance were derived for each individual. Curves were fit using the Levenberg–Marquardt residual minimization procedure, implemented in MATLAB R2018a (Natick MA, USA) using the inbuilt function nlinfit. To ensure we obtained accurate individual-level estimates of the ED50, we required that the 95% confidence interval for the individual-level fit span less than one log10-unit (i.e., a factor of 10) from minimum to maximum. This was satisfied in 42 of the participants.

#### Light monitoring study

##### Light metrics

Light exposure data were recorded by the wearable spectrophotometer device in 2-min epochs. Melanopic and photopic illuminance values were calculated onboard by the device for each epoch from the recorded spectrum using the approach described in the Irradiance Toolbox^17^. Values for melanopic illuminance were scaled by 0.9058 to be consistent with the latest CIE recommendations for melanopic daylight-equivalent illuminance^19^, which ensure an M:P ratio of 1 for a reference daylight source.

While participants were instructed to avoid coverage of the device, two cleaning rules were applied in cases where coverage was evident. First, epochs during wakefulness with very low illuminance (< 1 lux) were excluded. Second, epochs during wakefulness that had sudden temporary drops in illuminance were excluded if two rules were simultaneously satisfied: < 10 lux in the epoch, and < 10% of the illuminance in the 2-min epochs both immediately before and after. In total, 0.6% of evening epochs were removed by these cleaning rules.

##### Pre-bed light

For predicting melatonin suppression, we used individual-level dose-response curves in combination with home light recordings. Home light levels were averaged across the recorded nights within each individual, using the 3 h before bed (a time window selected to be centered on the 5-h time window used for the dose-response study). Light data were binned in 30-min windows (0–30 min before bedtime, 30–60 min before bedtime, etc.). Average melanopic illuminance was calculated by averaging across bins to ensure timepoints were equally weighted (i.e., not susceptible to bias due to variable amounts of missing data at different times relative to bedtime). We required that an individual had at least 50% of bin length (15 min) in total valid epochs for each bin across nights of recording to compute an average melanopic illuminance. The average melanopic illuminance was input to each of the individual-level dose-response curves to compute predicted melatonin suppression.

##### Light after sunset

To compare the variation in light levels to a natural sunset, we computed average light curves for each individual up to their average bedtime. A centered moving average with window length of 3 h was applied to each individual, averaging any timepoints within the clock-time range across nights of recording.

##### Sleep variables

We investigated whether melanopic illuminance in the 3 h before bedtime related to objective sleep quality, measured using actigraphy. Within each rest interval, Actiware (Philips Respironics, PA, USA) scored sleep vs. wake state in 1-min epochs, using a medium sensitivity wake threshold (40 activity counts). For each night, we computed percentage wakefulness in the first 90 min of the rest interval. A generalized linear mixed model, implemented in MATLAB R2018a (Natick MA, USA), was used to predict percentage wakefulness in each interval, including participants as random intercepts, and including fixed effects for age, sex, within-individual average (log-transformed) melanopic illuminance, deviation from average (log-transformed) melanopic illuminance, within-individual average bedtime, and deviation from average bedtime. As further analysis, we also included as fixed effects the Insomnia Severity Index, Pittsburg Sleep Quality Index, Morningness-Eveningness Questionnaire, and Epworth Sleepiness Scale.

##### Spectral classification

The recorded spectrum was averaged across the 3 h before bedtime for each night and interpolated to 1-nm resolution across the range 380–760 nm, then normalized by total area under the curve. We required at least 50% valid epochs (1.5 h of data) for a night to be included in this analysis. Heuristic metrics were used to classify spectra based on standard characteristics of common types of light sources. Incandescent light sources were detected by the characteristic increase in power towards red/infrared wavelengths, identified by linear slope > 6 × 10^–6^ in the range 440–760 nm. Sunlight was detected based on its broad spectrum, dividing the normalized spectrum (380–760 nm) into four equal wavelength ranges (95-nm width) and computing the area under each section of the curve. Sunlight was identified if the greatest absolute deviation in area from 25% was below 10%, and the average melanopic illuminance was above 20 mlux. The spectrum was then linearly detrended. Fluorescent light sources were detected by the presence of characteristic sharp spectral peaks, identified by the presence of steeply sloped sections in the range 520–620 nm, computed by $\sum\nolimits_{i} {\Delta S_{i}^{4} > {1}.{5} \times {1}0^{{ - {7}}} }$ where $\Delta S_{i}$ is the change in the normalized spectrum between successive points. LED light sources were detected using two key features of the spectrum: (1) decreasing linear slope ($m$) of the detrended spectrum in the range 640–760 nm, and (2) a peak detected in the blue wavelength range 430–470 nm, using the highest-order coefficient ($a$) of a quadratic fit to the linearly detrended spectrum in this range. A value of $m + 3.5a < - {1}.{5} \times {1}0^{{ - {5}}}$ was used to identify an LED source. Appropriate cut-offs were found by testing against standard CIE spectra. Spectra that satisfied the criteria for only one type of light source were labeled for analysis (LED, fluorescent, incandescent). Others were treated as hybrid/unclassified. Sunlight was detected in only two cases, both of which corresponded to early bedtimes (i.e., with the 3-h pre-bed window beginning before sunset), and both of which were classified as hybrid spectra due to detection of fluorescent light too. A linear mixed model was used to test for differences in melanopic illuminance and M:P ratio between light types, including light type as a fixed effect, and including individuals as random intercepts.

## Supplementary information

Supplementary Information.
