# Supplementary material for: Evening home lighting adversely impacts the circadian system and sleep
Source: Sci Rep. 2020 Nov 5;10:19110. doi: 10.1038/s41598-020-75622-4 (PMC7644684; doi:10.1038/s41598-020-75622-4)
Supplement: Supplementary file 1 — Supplementary Information. [file 41598_2020_75622_MOESM1_ESM.docx]

**Supplementary Material**

**Evening home lighting adversely impacts the circadian system and sleep**

# Sean W. Cain^1*^, Elise M. McGlashan^1^, Parisa Vidafar^1^, Jona Mustafovska^1^, Simon P. N. Curran^1^, Xirun Wang^1^, Anas Mohamed^2^, Vineetha Kalavally^2^, Andrew J. K. Phillips^1*^

^1^ School of Psychological Sciences and Turner Institute for Brain and Mental Health, Monash University, Melbourne, VIC, Australia

^2^ Department of Electrical and Computer Systems Engineering, School of Engineering, Monash University Malaysia, Bandar Sunway, 47500 Subang Jaya, Malaysia

**Table S1:** Within-individual variability in pre-bedtime light exposure is associated with poorer objective sleep quality, including additional covariates.

| **Wakefulness (minutes) in first 90 minutes after bedtime** | | | | | | |
| --- | --- | --- | --- | --- | --- | --- |
| **Within and between individual light, adjusted for age, sex, bedtime, chronotype, and subjective sleep quality** | | | | | | |
| Predictor | β | SE | t | Lower | Upper | p |
| Average light | -0.46 | 0.62 | -0.74 | -1.69 | 0.77 | 1.00 |
| Deviation from average light | 0.89 | 0.09 | 9.87 | 0.71 | 1.07 | **<.00001** |
| Age | -0.03 | 0.01 | -2.23 | -0.05 | 0.00 | **.03** |
| Female Sex | -0.54 | 0.30 | -1.80 | -1.14 | 0.05 | .07 |
| Average bedtime | 0.50 | 0.48 | 1.04 | -0.44 | 1.44 | .30 |
| Deviation from average bedtime | -0.01 | 0.03 | -0.51 | -0.07 | 0.04 | .61 |
| MEQ | 0.01 | 0.02 | 0.53 | -0.02 | 0.04 | .59 |
| PSQI | 0.15 | 0.06 | 2.44 | 0.03 | 0.28 | **.02** |
| ESS | 0.02 | 0.05 | 0.46 | -0.07 | 0.11 | .65 |
| ISI | 0.00 | 0.05 | 0.05 | -0.10 | 0.10 | .96 |

Generalized linear mixed model for minutes of wakefulness in the first 90 minutes after bedtime, using a binomial distribution and logit link function. Predictors included age (years), sex, average light (log-transformed melanopic illuminance in 3 h before bedtime), deviation from average light (using log-transformed values), average bedtime (decimal hours), deviation from average bedtime (hours), Morningness-Eveningness Questionnaire (MEQ) score, Pittsburgh Sleep Quality Index (PSQI) score, Epworth Sleepiness Score (ESS), and the Insomnia Severity Index (ISI). The table shows unstandardized coefficients (β), standard errors (SE), t value, Lower and Upper 95% confidence intervals, and p-valued (significant values in bold).


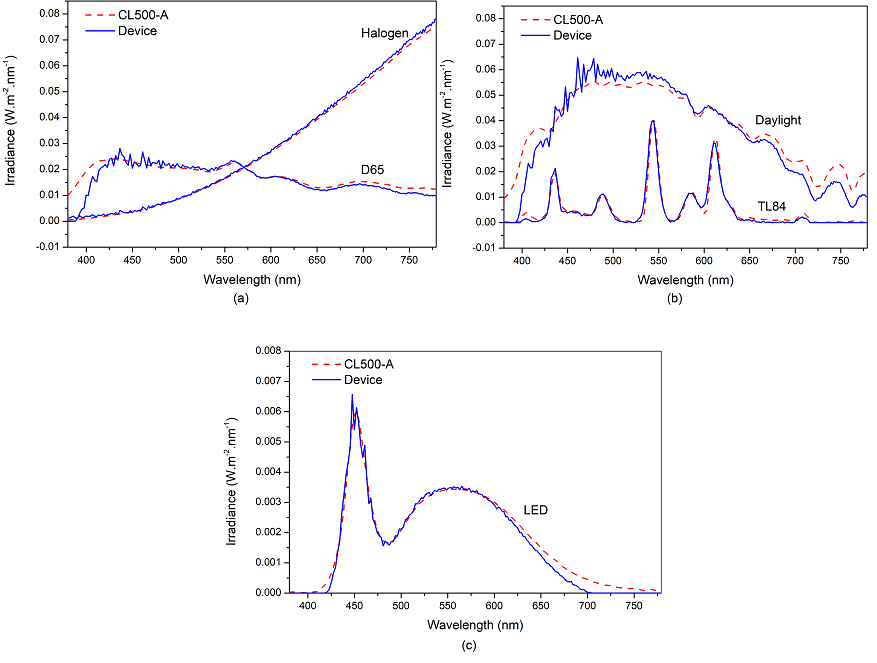


**Figure S1:** Absolute irradiance measurements, comparing the light pin device with the CL500-A standard spectrophotometer. Illuminant sources used were: (a) D65 and Halogen; (b) TL84 Fluorescent and Daylight; and (c) LED.
